# Ethical Challenges and Current Opportunities of Artificial Intelligence in Cardiology

**DOI:** 10.1055/s-0045-1809879

**Published:** 2025-06-20

**Authors:** Mohammed A.R. Chamsi-Pasha, Hassan Chamsi-Pasha

**Affiliations:** 1 Department of Cardiovascular Medicine, Houston Methodist Hospital, Houston, Texas, United States; 2 Department of Cardiology, European Medical Center, Jeddah, Saudi Arabia

**Keywords:** artificial intelligence, cardiovascular disease, ethics, ChatGPT

## Abstract

Artificial intelligence (AI) has great potential in diagnosing, managing, and predicting cardiovascular diseases through imaging, clinical decision support, remote monitoring, and optimizing treatment strategies. AI in cardiology brings unique ethical issues that need careful examination and resolution. There are several ethical concerns, including privacy, bias, trust, accountability, and responsibility. AI systems handle large quantities of data, which can present privacy and security risks if hacked or exploited illegally. AI models may exhibit biases due to limited or nonrepresentative training data sets, impacting their reliability. ChatGPT shows potential in cardiology for patient education, clinician support, and research facilitation. However, its use in direct patient care is limited due to concerns regarding accuracy, ethical issues, and the necessity for human oversight. AI's responsible development and application in cardiology hinges on thorough evaluation, regulatory compliance, and ethical oversight to ensure safety and effectiveness. The collaboration of health care professionals, data scientists, ethicists, researchers, and policymakers is essential for the advancement of AI in cardiology and the resolution of its associated challenges. Collaboration is mandatory to ensure AI tools improve patient care while upholding the highest medical standards. The incorporation of AI into cardiology offers significant potential for the coming years. With its extensive data sets and strong evidence-based guidelines, cardiology is ideally suited to using this technology.

## Introduction

Cardiovascular diseases (CVDs) are the leading cause of death worldwide, underscoring the importance of prompt and precise diagnosis. Artificial intelligence (AI) and machine learning (ML) have become crucial tools in the health care sector, offering substantial potential for cardiovascular diagnosis and imaging advancements, revolutionizing patient care, research, and clinical decision-making. 1

Deep learning algorithms are currently employed to analyze computed tomography (CT) and magnetic resonance imaging (MRI) studies, predict the results of exercise tests, and forecast the outcomes of interventional procedures like transcatheter valve replacements. AI-assisted electrocardiography (ECG) analysis enhances arrhythmia detection, heart failure risk stratification, and myocardial infarction diagnosis. ML models optimize cardiac catheterization reports, coronary angiograms, and telemedicine applications. AI models can identify novel genetic associations with cardiac conditions, aiding in personalized medicine. 2 3

The AI algorithms can predict cardiovascular risk with greater accuracy than physicians, aiding in the customization of management plans, and enhancing patient medication adherence and health care engagement.

The swift progress of AI in cardiology offers promising opportunities but also presents significant challenges that require thorough evaluation. Implementing AI in cardiovascular medicine should be based on core bioethical principles: autonomy, beneficence, nonmaleficence, and justice. The literature highlights several significant ethical issues, including bias, privacy, trust, accountability, and responsibility. 4 5

## Clinical Applications

Recent advances focus on improving imaging techniques, disease detection, and risk prediction using ML and deep learning models. These technologies offer insights that are often beyond the reach of clinicians, particularly in fields such as imaging and ECG analysis. 6

ML can analyze images from echocardiography, cardiac MRI, and CT angiography. These algorithms can identify subtle differences and uncover hidden signals that are not visible to the human eye. 7

AI has the potential to revolutionize traditional diagnostic tools by converting them into advanced predictive instruments. These tools can identify nuance changes in ECGs that may indicate future cardiovascular events. AI can predict new cases of atrial fibrillation (AF), detect systolic heart failure, and diagnose heart attacks in the emergency room. AI can improve decision-making in urgent situations, optimize resource distribution, and tailor treatment plans. 8 9

AI models outperform traditional ECG interpretation in detecting conditions such as reduced ejection fraction, cardiomyopathies, and valvular heart disease. Deep learning techniques significantly enhance arrhythmia classification and can predict AF risk from normal sinus rhythm. AI-powered smartwatches and wearable devices enable early detection of AF, heart failure decompensation, and abnormal rhythms. 3 AI-supported ECG screening has effectively detected hypertrophic cardiomyopathy and cardiac amyloidosis early. 10 11

The Mayo Clinic has examined over 650,000 ECGs from patients with various heart conditions. This extensive analysis has enabled the creation of AI/ML algorithms that provide valuable clinical insights. For instance, these algorithms can identify a patient's gender, estimate their biological age, and detect signs of left ventricular dysfunction using just a few heartbeats. The ECG algorithm may detect previous episodes of AF in patients who are in sinus rhythm during the examination. 6

ML algorithms can greatly enhance risk assessment and assist in treatment choices for conditions such as long QT syndrome, Brugada syndrome, and hypertrophic cardiomyopathy. They can predict the risk of arrhythmias with higher accuracy compared with traditional risk scores. 12 In acute myocardial infarction, the latest generation of AI demonstrated superior performance compared with traditional ST-elevation myocardial infarction criteria, offering twice the sensitivity while maintaining the same level of specificity. 13

An important area where AI can make notable progress is in diagnostic imaging. Several studies have demonstrated that AI can significantly improve the precision, speed, and accuracy of various imaging techniques such as echocardiography, CT, and MRI.

In echocardiography, AI applications assist with tasks like ventricular segmentation, measuring ejection fraction, and analyzing wall motion. Advances in deep learning and large-scale computing have made it possible to quickly acquire parameters and predict diseases and cardiovascular events, with or without quantitative data. An automatic echocardiography report generation is anticipated. 3 14 15

AI-enhanced coronary CT angiography has been utilized to detect and quantify atherosclerotic plaques, offering a precise and efficient characterization of coronary artery disease (CAD). Recent advancements in AI have led to the development of models that can automatically detect cardiac anomalies, significantly enhancing diagnostic accuracy and reducing operator variability.

Cardiac magnetic resonance (CMR) has also seen advancements due to AI innovations, especially in functional MRI, which allows for more detailed tissue analysis. AI can transform CMR imaging from acquisition to diagnosis. AI quantifies CMR images, assessing strain, myocardial motion, and perfusion. 1 10

AI enhances intracardiac signal analysis to guide electrophysiology procedures, such as AF ablation. ML models assist in coronary artery stenosis detection and provide real-time decision support during catheterization. 10

AI improves preoperative risk assessment, intraoperative decisions, and postoperative care by offering better risk stratification and more accurately predicting complications like AF or acute kidney injury than traditional methods. 16 Robotically assisted percutaneous coronary intervention is expected to enhance angiography's precision and lower the proceduralist's occupational hazards. AI refines genetic risk stratification by identifying pathogenic variants associated with CVDs. AI models predict future cardiovascular events by analyzing electronic health records (EHR), laboratory results, and imaging data. 10 Integrating EHR data with AI enhances CVD risk prediction and management. 17

We are currently witnessing the emergence of foundation models in medical AI, which are large, adaptable systems trained on extensive and diverse data sets, offering superior performance compared with traditional task-specific models. It is crucial to thoroughly validate, carefully implement, and continuously evaluate AI tools used in clinical practice. 4

## ChatGPT

The development of AI, particularly large language models such as ChatGPT, creates new possibilities for preventing, diagnosing, managing, and researching CVD. ChatGPT is revolutionizing health care by reducing administrative tasks, enhancing medical education, and aiding research with literature reviews and hypothesis generation.

ChatGPT improves medical education by providing personalized learning, automated scoring, and instant access to extensive medical knowledge. 18 ChatGPT-based models may have potential in ECG interpretation, but they currently lack adequate reliability. Clinical use of ChatGPT requires more validation, regulatory oversight, and continuous improvements in model accuracy and reliability. 19 By offering clear and understandable explanations of cardiovascular conditions, procedures, and lifestyle changes, ChatGPT can assist patients in managing their health. 20

ChatGPT has limitations in cardiology; it uses preexisting data sets, so it may lack current medical recommendations. AI-generated responses can sometimes be error-prone generating incorrect citations, a phenomenon referred to as “hallucination” or “stochastic parroting.” 20 21

Other chatbot systems capable of natural language conversations using advanced language models include Google Bard, Microsoft Copilot, DeepSeek, Claude, and Perplexity.

## Ethical Challenges

### Equity

According to the World Health Organization, “equity” means eliminating avoidable, unfair, or fixable differences among various social, economic, demographic, or geographic groups. To reach the goal of health care equity, it is essential to use data sets that represent the entire population when developing AI/ML algorithms. Additionally, prioritizing equity should be explicitly mentioned as a goal during the creation of these health care AI/ML tools. AI in health care should be made widely accessible and should be developed to ensure its use across all ages, races, genders, incomes, ethnicities, and locations. 22

### Bias

AI algorithms need extensive data for training and to develop an understanding of a problem based on the data provided. If the data are limited or inaccurate, it will lead to biased models. Nolin-Lapalme et al classified biases in AI models related to cardiology into four types: data bias, algorithm bias, assessment bias, and user bias. 23 Statistical bias arises from nonrepresentative samples in the training data, such as undersampling or excluding certain populations. If a data set primarily consists of certain demographics, such as middle-aged Caucasian males, the AI model may not perform effectively for other groups. This lack of diversity can lead to biased outcomes and reduced accuracy in diagnosing and treating these underrepresented races and ethnicities that experience the worst health outcomes. 22 Low- and middle-income countries bear approximately 80% of the global CVD burden. Favoring data from Western European or North American sources over data from Asian or African countries will inherently lead to biased algorithms. AI tools in cardiovascular medicine frequently necessitate sophisticated infrastructure, advanced imaging technologies, or wearable devices, which might be unavailable in resource-limited regions. Most studies on these devices come from high-income countries, which raise ethical and safety concerns. 24 AI-powered CAD risk calculators might not work in low-income areas without proper infrastructure. Another important issue is that most AI developers are men, potentially disadvantaging women. Despite women with AF having higher mortality risks, only 29% of smartwatch users are women. 24

Proposed strategies to tackle these challenges include meticulous data curation, development of algorithm design techniques to reduce bias, and the promotion of transparency in AI research. 23

### Privacy and Consent

Creating AI systems necessitates extensive access to detailed patient data for algorithm training. The large volume of data makes sensitive information susceptible to cyber threats. It is essential to have clear safeguards in place. In cardiology, AI frequently utilizes patient health records, imaging data, and real-time information from wearable devices such as smartwatches. Robust legal frameworks are essential to ensure that identities are anonymized and safeguarded against breaches.

AI systems should not access patient data without obtaining informed and valid consent. Patients should be informed about how their data are being used whether it is identified or deidentified. 4 22 Rose and Shapiro recommended evaluating AI use cases based on specific criteria to determine the proper category (no notification or no informed consent [IC], notification only, and formal IC): (1) the level of AI model autonomy, (2) the extent to which the AI deviates from standard practices, (3) whether the AI model interacts directly with patients, (4) the clinical risks associated with the model, and (5) the administrative burdens involved. 25

While the default ethical stance requires IC for using personal health data, exceptions exist under regulated conditions. Nonetheless, prioritizing transparency and obtaining consent whenever possible remains the best practice to uphold ethical standards in health data usage. Individuals should not encounter limitations on medical treatment or essential services if they decide to withhold consent. Additionally, organizations should not provide incentives to individuals to obtain consent, such as insurance companies offering wearable technology to customers in exchange for access to their health data. 26

### Liability

Evaluating the liability of AI/ML algorithms is essential to appropriately balance their associated risks and advantages. AI systems can give incorrect or unsafe recommendations, like misidentifying benign arrhythmias as critical. It is unclear who is responsible for AI errors: developers, clinicians, or health care institutions. The integration of AI into clinical practice raises pressing concerns, paralleling ethical and legal debates seen with autonomous vehicles—particularly regarding accountability. In instances where an AI system provides guidance that a clinician chooses to override, and adverse outcomes occur, the question of responsibility becomes complex. Addressing such issues is essential prior to the widespread adoption of AI in health care, with the answers likely varying based on the type and application of the AI tool involved. 7

In their scoping review, Bouhouita-Guermech and Haidar analyzed 136 articles. The findings revealed an absence of a well-defined framework for assigning responsibility in the use of AI within health care. They also highlighted the critical need for the ethical and accountable development and deployment of AI technologies in this field. 27

A Scientific Statement from the American Heart Association suggested that companies should apply to the Food and Drug Administration (FDA) for approval to market an algorithm. After approval, postmarket safety monitoring should be conducted similarly to phase IV drug evaluations. During this phase, if the algorithm's use results in adverse events or system failures, the developers are responsible for reporting and investigating these outcomes. Consequently, a physician's liability in the event of an incorrect decision and potential harm, as with any medical product, is limited to ensuring the algorithm is used as “labeled,” thereby minimizing liability concerns. 22 While clinicians are expected to use AI/ML algorithms in accordance with their intended use, it is still unclear whether they will be held legally accountable for any harm resulting from these tools. Additionally, it remains uncertain whether the integration of such technologies will redefine the standard of care in clinical practice.

Nonmedical staff can be trained quickly to conduct basic cardiac procedures such as echocardiography and pacing device interrogation. However, the responsibility for the accurate execution and reporting of these cardiac investigations should remain with the qualified clinician. 2

## Regulation and Oversight

The advancement of AI in cardiology is progressing quickly, sometimes faster than regulatory bodies can evaluate and approve these tools. Insufficient oversight could result in the early deployment of technologies that have not been fully tested, potentially increasing patient risk. It is important for AI tools to undergo thorough validation on diverse, real-world data sets. Just as computer-assisted ECG analysis still requires cardiologist oversight, expert clinicians must remain involved to prevent potential AI-related harm. 3

The use of AI in clinical decision-making must align with privacy laws and ethical guidelines. AI deployment requires adherence to FDA guidelines, patient data protection laws (Health Insurance Portability and Accountability Act, General Data Protection Regulation), and ethical AI governance.

## Transparency and Explainability

In health care, transparency means being able to access information about how AI and ML algorithms work and make decisions. Explainability is about ensuring stakeholders can understand and interpret the results from these algorithms in a clear way. 28

A frequent critique of utilizing AI in health care is sophisticated technology's “black box” characteristic. This term describes the perceived lack of understanding among health care providers about AI systems' development, reliability, testing, and potential issues. If an AI system predicts a high risk of arrhythmia, for example, the clinician may not understand which features contributed to the prediction.

Creating explainable AI is a key focus for policymakers to foster trust in new systems. Offering a detailed explanation of how complex algorithms work is challenging for intricate ML models. A certain degree of comprehension of these algorithms may assist patients in giving IC for using AI in their medical treatment.

Outputs produced by AI systems should be clearly labeled as such and, when possible, supplemented with explanatory information. 3 Ensuring transparency and providing understandable reasoning behind AI and ML recommendations are essential to fostering trust among health care professionals and patients, thereby supporting informed clinical decision-making. 2 28 Patients may be reluctant to trust AI-derived recommendations if they view the system as impersonal or prone to errors. It is crucial to ensure that patients comprehend how AI assists in their care, while also highlighting its limitations. Understanding AI system accountability, fairness, transparency, and explainability is paramount for user trust.

## Conclusion

AI is transforming cardiovascular care by enhancing diagnostics, treatment strategies, and patient outcomes. Recent advances focus on improving imaging techniques, disease detection, and risk prediction using ML and deep learning models. AI-driven solutions now extend across ECG, echocardiography, CMR, nuclear cardiology, coronary angiography, and others.

AI has surpassed traditional methods in many areas but remains limited by the need for extensive labeled data sets, potential model overfitting, and transparency and bias issues. While AI has the potential to enhance human intelligence, it is unlikely to serve as a complete substitute. Human qualities such as creativity, adaptability, and critical thinking remain indispensable, particularly in complex domains like scientific inquiry and various aspects of daily life.

Several ethical concerns regarding the use of AI in cardiology need to be addressed. A multidisciplinary approach is essential, involving: Transparent AI development processes, robust regulatory frameworks, ongoing education for clinicians and patients, ensuring data sets represent all demographic groups, and establishing clear guidelines for developing, validating, and deploying AI tools. Organizations such as the World Health Organization, the U.S. FDA, and the American Medical Informatics Association have emphasized the need to incorporate ethical principles into the development and use of emerging technologies. By addressing these ethical issues through policies, collaboration, and research, AI can be integrated into cardiology to improve care while adhering to ethical standards.
